# Acute kidney injury is associated with a decrease in cortical renal perfusion during septic shock

**DOI:** 10.1186/s13054-018-2067-0

**Published:** 2018-06-15

**Authors:** Anatole Harrois, Nicolas Grillot, Samy Figueiredo, Jacques Duranteau

**Affiliations:** Anesthesia and Intensive Care Department, Hôpitaux Universitaires Paris-Sud, Université Paris-Sud, Université Paris-Saclay, Hôpital De Bicêtre, Assistance Publique Hôpitaux de Paris (APHP), 78, Rue du Général Leclerc, 94270 Le Kremlin-Bicêtre, France

**Keywords:** Acute kidney injury, Renal failure, Renal perfusion, Sepsis, Septic shock, Renal ultrasonography

## Abstract

**Background:**

Renal perfusion status remains poorly studied at the bedside during septic shock. We sought to measure cortical renal perfusion in patients with septic shock during their first 3 days of care using renal contrast enhanced ultrasound (CEUS).

**Methods:**

We prospectively included 20 ICU patients with septic shock and 10 control patients (CL) without septic shock admitted to a surgical ICU. Cortical renal perfusion was evaluated with CEUS during continuous infusion of Sonovue (Milan, Italy) within the first 24 h (day 0), between 24 and 48 h (day 1) and after 72 h (day 3) of care. Each measurement consisted of three destruction replenishment sequences that were recorded for delayed analysis with dedicated software (Vuebox). Renal perfusion was quantified by measuring the mean transit time (mTT) and the perfusion index (PI), which is the ratio of renal blood volume (rBV) to mTT.

**Results:**

Cortical renal perfusion was decreased in septic shock as attested by a lower PI and a higher mTT in patients with septic shock than in patients of the CL group (*p* = 0.005 and *p* = 0.03). PI values had wider range in patients with septic shock (median (min-max) of 74 arbitrary units (a.u.) (3–736)) than in patients of the CL group 228 a.u. (67–440)). Renal perfusion improved over the first 3 days with a PI at day 3 higher than the PI at day 0 (74 (22–120) versus 160 (88–245) *p* = 0.02). mTT was significantly higher in patients with severe acute kidney injury (AKI) (*n* = 13) compared with patients with no AKI (*n* = 7) over time (*p* = 0.005). The PI was not different between patients with septic shock with severe AKI and those with no AKI (*p* = 0.29).

**Conclusions:**

Although hemodynamic macrovascular parameters were restored, the cortical renal perfusion can be decreased, normal or even increased during septic shock. We observed an average decrease in cortical renal perfusion during septic shock compared to patients without septic shock. The decrease in cortical renal perfusion was associated with severe AKI occurrence. The use of renal CEUS to guide renal perfusion resuscitation needs further investigation.

## Background

Renal function impairment is a frequent condition during septic shock with an incidence of acute kidney injury (AKI) ranging from 55 to 73% [1–3]. It is independently associated with mortality [4]. Renal perfusion alterations, especially at the microvascular level, appear to play a crucial role in AKI occurrence [5]. However, renal perfusion status remains poorly studied at the bedside during septic shock and macrovascular parameters (i.e. mean arterial pressure (MAP) and systemic oxygen delivery) mostly guide hemodynamic resuscitation in the intensive care unit (ICU) without renal microcirculation monitoring, which is often unavailable at the bedside in daily practice. Thus, despite optimization of macrovascular hemodynamic parameters according to Surviving Sepsis Campaign guidelines [6], occult renal hypoperfusion may persist with an early onset of renal dysfunction [7]. On the other hand, after initial hemodynamic resuscitation, renal blood flow (RBF) was also reported to be normal or even increased during septic shock with AKI developing concurrently [7, 8]. Since renal function impairment may also occur despite a preserved global RBF, the study of intrarenal blood flow distribution becomes an important step in understanding the pathophysiology of AKI during septic shock. Hence, whether alterations of microvascular RBF are due to insufficient hemodynamic optimization or to specific microvascular injuries induced by sepsis (endothelial dysfunction, increased leukocyte adhesion, rheological abnormalities, glycocalyx alterations and functional shunting), we crucially need a tool to measure renal perfusion in daily practice [5, 9, 10].

Several tools were proposed to investigate renal microcirculatory perfusion at the bedside. Renal Doppler ultrasound measures the variations in intrarenal resistances and was proposed to assess a modification in renal perfusion after fluid loading [11, 12] or vasopressor administration [13]. However, it does not directly quantify renal microcirculatory perfusion. Renal contrast-enhanced ultrasound (CEUS) was recently proposed to quantify renal cortical perfusion at the bedside in various conditions like chronic kidney disease, angiotensin intravenous infusion or captopril oral intake [14, 15]. During CEUS, the use of intravenous contrast product increases blood echogenicity and enhances the visualization of microcirculatory renal perfusion when using ultrasonography. Good correlation has been demonstrated between CEUS measurements of renal perfusion and gold standard renal blood flow measurements (para-aminohippurate clearance) [14]. Experimental data on isolated pig and dog kidney reveals that renal cortical CEUS differentiates between macro and microcirculation [16, 17]. This promising tool has been used in ICU patients in two pilot studies [18, 19].

Since we lack clinical data on renal microcirculatory perfusion during septic shock, we intended to measure renal cortical perfusion with CEUS during the first 3 days in patients with septic shock.

The aims of our study were:

1. To compare cortical renal perfusion between patients with septic shock and ICU patients without sepsis
2. To evaluate renal cortical perfusion in patients with septic shock who develop or do not develop AKI
3. To test correlation between renal cortical perfusion and macrohemodynamic and microhemodynamic data

## Methods

### Patients

This study was approved by the comité d’Evaluation de l’Ethique des projets de Recherche Biomédicale (CEERB) du Groupe Hospitalier Universitaire (GHU) Nord (Institutional Review Board (IRB) of Paris North Hospitals, Paris 7 University, AP-HP) with the number 11-065. The institutional Review Board waived the need for informed consent. Patients (or relatives) were informed (explanatory note) and we obtained confirmation of non-objection to data use.

Patients were recruited in the 28-bed surgical ICU of the Bicêtre University Hospital between January 2014 and July 2015. Inclusion criteria were septic shock defined by the presence of two or more diagnostic criteria for systemic inflammatory response syndrome: elevated heart rate (> 90/min), tachypnea > 20/min, hyperthermia/hypothermia (> 38.3 °C/< 36 °C), leucocytosis or leukopenia (> 12,000/mm^3^ or < 4000/mm^3^), proven infection and vasopressors (norepinephrine or epinephrine) at a minimum rate of 0.1 μg.kg^− 1^.min^− 1^.

Exclusion criteria were pregnancy, age < 18 years, urologic sepsis, left ventricular ejection fraction < 30% (measured with transthoracic echocardiography), systolic pulmonary arterial pressure > 90 mmHg (measured with transthoracic echocardiography), recent ischemic heart disease (within < 7 days) and chronic hemodialysis.

A control (CL) group constituted 10 ICU patients with brain damage (traumatic brain injury, stroke) without septic shock with an expected ICU length of stay > 72 h. These control patients were enrolled during the same period as patients with septic shock.

### Study design

Renal perfusion was measured using CEUS at study inclusion, which was considered to be day 0 (within the first 24 h of ICU hospitalization), and at day 1 and day 3. All the measurements were acquired in patients with stable hemodynamics (i.e. MAP ≥ 65 mmHg with a stable norepinephrine infusion over 2 h). Demographic data and Simplified Acute Physiology Score (SAPS) II were reported at admission. The Sequential Organ Failure Assessment (SOFA) score, heart rate and MAP were reported. Cardiac index was measured by transthoracic echocardiography (2–4 MHz probe) using the Aplio 500 ultrasound machine (Toshiba Medical Systems Co. Ltd., Otawara, Japan) as described elsewhere [20]. Serum lactate, blood gases, norepinephrine infusion rate, serum creatinine, urine creatinine, serum urea, 24 h diuresis and 8 h diuresis (4 h before and 4 h after each CEUS measurement) were reported at day 0, 1 and 3. The severity of AKI was evaluated by the Kidney Disease Improving Global Outcomes (KDIGO) criteria. The renal resistivity index (RI) was measured in the interlobar arteries using Doppler ultrasound (Aplio 500, Toshiba Medical Systems Co. Ltd., Otawara, Japon) with a 6–11 MHz transducer. The Doppler spectrum was considered optimal when three consecutive waveforms were obtained. The RI was calculated as follows:

(Systolic velocity – End diastolic velocity)/Systolic velocity.

### Contrast enhanced ultrasound measurements

The Sonovue® (Bracco, Milano, Italy) contrast ultrasound agent was used for this study. After reconstitution, the contrast ultrasound agent was diluted with an equal amount of NaCl 0.9%. It was infused through a central venous catheter with a dedicated Vueject® syringe pump (Bracco, Milano, Italy) at a rate of 1 mL.min^− 1^ all along the sequence acquisition. The left kidney was visualized in B mode in its long axis view with an abdominal convex probe (6–11 MHz, Toshiba Medical Systems Co. Ltd., Otawara, Japon). Synchronous images were acquired in contrast ultrasound mode. The same echo parameters were applied for each acquisition to allow for contrast intensity comparison:

Mechanical index = 0.07 – Depth = 12 cm – Gain = 73 – Frame rate = 10 s^− 1^.

After 2 min of continuous contrast ultrasound agent administration (steady state), three consecutive destruction/replenishment sequences (with 15 s replenishment) were acquired. The destruction phase consisted of an ultrasound flash of high mechanical index (mechanical index = 1) that instantaneously destroyed the contrast ultrasound agent in the ultrasound plane observed with the probe. The replenishment phase corresponds to the progressive contrast enhancement of the kidney ultrasound plane after destruction.

### CEUS sequences analysis

CEUS was performed at day 0, day 1 and day 3 in each patient. The ultrasound sequence was exported in digital imaging and communication in medicine (DICOM) format. Renal perfusion was analyzed using VueBox v 4.3 software (Bracco, Research, Geneva, Switzerland). The region of interest (ROI) was the cortical zone of the kidney parenchyma. Movement compensation (because of respiratory movements of the kidneys) was automatically applied. DICOM data on the ROI were converted into echo power data that are proportional to the concentration of the ultrasound contrast product in the parenchyma. A time-intensity curve (intensity of renal parenchyma replenishment over time) of the ROI was generated and three analysis parameters were calculated from this curve:

- mTT (mean transit time, seconds) is the time needed after contrast agent destruction to reach 50% of the maximal intensity signal
- rBV (renal blood volume, a.u.) is a measure of the ROI maximal intensity after complete replenishment
- PI (perfusion index) is calculated by dividing rBV by mTT

The values obtained for the three destruction/replenishment sequences were averaged for each time measurement (day 0, day 1 and day 3).

### Statistical analysis

Data are expressed as mean ± standard deviation or median (interquartile range) unless otherwise specified. Categorical data are expressed as number (percentage). Comparisons of general characteristics between septic shock group and CL at baseline (day 0) were performed using the Student t test or the Mann Whitney test according to the type of data. Comparison of proportions were done using Chi-squared test.

The evolution of CEUS parameters (mTT, PI) over time in septic shock group was evaluated with a one factor ANOVA on ranks (factor time: day 0, day 1, day 3). A *post hoc* analysis with a Bonferroni correction was done between days if the global ANOVA was significant.

Evolution of CEUS parameters (mTT, PI, rBV) over time was compared between the septic shock group and CL or between patients with severe AKI (AKI stage 2 or 3) or no AKI (no AKI or AKI stage 1) during the first 72 h in patients with septic shock or between ICU survivors and ICU non survivors in patients with septic shock with a two way ANOVA analysis on ranks: a factor group and a factor time (day 0, day 1, day 3).

We plotted a receiver operating characteristic (ROC) curve of sensitivity versus (1-specificity) for various threshold values of a variable (mTT, PI, cardiac index, Doppler RI) at day 0 and calculated the area under this ROC curve to evaluate whether these variables may discriminate patients according to the occurrence of severe AKI (KDIGO stage 2 or 3).

Three CEUS measurements were obtained in the 30 patients. Thus, 90 measurements should have been done; however, 6 measurements were not be obtained in 4 patients because they died before day 3, and 2 other measurements were not obtained in a further 2 patients because of ultrasound system unavailability. The missing values in these six patients would have modified the strength of the statistical analysis. A value of zero could have been attributed to the missing data on the patients who died; however, it would have increased the difference between groups. That is the reason why we chose a more conservative approach by attributing the same value as the previous measure to missing data. We used the same approach with the other two patients who had missing data because of ultrasound system unavailability.

A *p* value <0.05 was considered to be statistically significant. The statistical analysis was conducted with software Prism (GraphPad software) and R 3.1.1 (http://www.R-project.org).

## Results

### General characteristics of the patients

We enrolled 20 patients with septic shock and compared their CEUS data with those observed in 10 sedated and ventilated patients without septic shock. Nine patients died in the septic shock group before day 28. Demographics and baseline physiology at day 0 are reported in Table 1. The source of infection was mainly surgical (peritonitis and fasciitis, *n* = 16/20). MAP was significantly lower in patients with septic shock (73 (67–80) mmHg) than in control patients (98 (94–108) mmHg) at baseline (*p* <  0.001). Norepinephrine infusion rate, serum lactate and creatinine values were significantly greater in the septic shock group than in the control group. No difference in cardiac index and urine output was found between the septic shock group and the control group at baseline (day 0). SAPS II and SOFA scores were significantly lower in control patients than in patients with septic shock (*p* = 0.001 and *p* = 0.003, respectively). Among patients with septic shock, 65% (*n* = 13/20) had severe AKI (KDIGO stage 2 or 3) during the first 72 h (Table 2). Severe AKI occurred early, as patients had evolving or established AKI at day 0 (first 24 h), with one patient evolving from stage 1 (day 0) to stage 2 (day 3) and 6 patients evolving from stage 2 (day 0) to stage 3 (day 1 or 3) over the first 3 days of care. Daily creatinine is reported in Table 3. None of the patients of the control group had severe AKI (KDIGO stage 2 or 3).

**Table 1:** General characteristics of the septic shock and the control groups

Variables		Septic shock (*n* = 20)	Control (*n* = 10)	*p*
Age, years		71 (51–84)	50 (42–58)	0.02
Ratio, male/female		12/8	8/2	0.27
BMI, kg.m^− 2^		27 (22–31)	25 (22–29)	0.75
SAPS II		47 (59–75)	38 (31–44)	0.001
SOFA		8 (7–11)	5 (4–6)	0.003
Pathology, *n*	Peritonitis	13	–	
	Pneumonia	3	–	
	Fasciitis	3	–	
	Arthritis	1	–	
	ICH	–	5	
	TBI	–	4	
	Stroke	–	1	
MAP, mmHg		73 (67–80)	98 (94–108)	< 0.001
Heart rate, min^− 1^		103 (88–122)	86 (67–106)	0.04
Norepinephrine, μg.kg^− 1^.min^− 1^		0.22 (0.13–0.53)	0.02 (0–0.3)	0.03
Epinephrine, μg.kg^− 1^.min^− 1^		0 (0–0.05)	0 (0–0)	0.14
Cardiac index, L.min^−1^.m^− 2^		2.4 (2–3.2)	2.1 (2.1–3.2)	0.31
Urine output, mL.kg^− 1^.h^− 1^		0.4 (0.3–1.1)	0.8 (0.3–1.4)	0.29
Lactate, mmol.L^−1^		2.6 (2–3.9)	1.3 (0.9–1.6)	< 0.001
Urea, mmol.L^−1^		14.1 (9.4–18.5)	6 (4–8)	0.001
Creatinine, μmol.L^−1^		152 (110–229)	57 (51–77)	< 0.001
PaO_2_/FiO_2_ ratio		212 (160–276)	339 (270–416)	0.007
Doppler resistivity index		0.74 (0.68–0.79)	0.66 (0.55–0.74)	0.06
Mortality at 72 h, *n* (%)		4 (20)	2(20)	0.23
ICU mortality, *n* (%)		9 (45)	3 (30)	0.43

*BMI* body mass index, *ICH* intracranial hemorrhage, *MAP* mean arterial pressure, *SAPS II* Simplified Acute Physiology Score, SOFA Sequential Organ Failure Assessment, *TBI* trauma brain injury; *PaO*_*2*_*/FiO*_*2*_ partial pressure of arterial oxygen/fraction of inspired oxygen

**Table 2:** Acute kidney injury occurrence in septic shock group according to KDIGO criteria

KDIGO criteria	No AKI	Stage 1	Stage 2	Stage 3	AKI-	AKI+
Creatinine, *n* (%)	6	2 (10)	5 (25)	7 (45)	8	12
Oliguria, *n* (%)	7	2 (10)	4 (20)	7 (35)	9	11
Creatinine and/or oliguria, *n* (%)	5	2 (10)	4 (20)	9 (45)	7	13

Patients are classified according to the most severe stage during the first 72 h of care. *KDIGO* Kidney Disease Improving Global Outcomes, *AKI* acute kidney injury, *AKI-* no AKI or AKI stage 1, *AKI+* AKI stage 2 or 3

**Table 3:** Macrohemodynamic and microhemodynamic variables in patients with septic shock with severe acute kidney injury and patients with septic shock with no acute kidney injury

Variables	Day 0	Day 1	Day 3
MAP, mmHg			
AKI+	67 (65–77)	79 (65–87)	81 (72–87)
AKI-	78 (74–86)	88 (77–95)	87 (77–93)
Cardiac index, L.min^−1^.m^−2^			
AKI+	2.4 (2.0–2.7)	2.9 (2.4–3.1)	2.5 (2.0–2.8)
AKI-	3.1 (2.1–3.9)	3.0 (2.2–3.5)	3.2 (2.3–4.0)
Norepinephrine, μg.kg^−1^.min^− 1^			
AKI+	0.25 (0.16–0.49)	0.45 (0.05–0.72)	0.03 (0.0–0.11)
AKI-	0.18 (0.11–0.76)	0.0 (0.0–0.09)	0.0 (0.0–0.0)
Lactate, mmol.L^−1^			
AKI+	3.7 (2.5–4.2)	2.6 (1.6–3.8)	1.6 (1.0–1.8)**
AKI-	1.9 (1.3–2.1)	1.4 (1.05–1.65)	1.5 (0.6–1.6)
Creatinine, μmol.L^−1^			
AKI+	186 (154–251)	187 (120–249)	130 (74–216)
AKI-	84 (68–120)	50 (41–82)	43 (40–63)
Doppler resistivity index			
AKI+	0.76 (0.70–0.81)	0.77 (0.70–0.84)	0.74 (0.68–0.85)*
AKI-	0.72 (0.67–0.75)	0.64 (0.61–0.70)	0.61 (0.59–0.74)
Renal CEUS variables			
PI			
AKI+	58 (20–86)	64 (31–145)	124 (35–235)
AKI-	100 (70–184)	195 (158–344)	230 (125–378)
mTT			
AKI+	5.6 (4.9–6.6)	5.3 (3.8–6.8)	5.5 (3.6–7.1)§§
AKI-	3.4 (3.1–3.8)	3.0 (2.6–3.9)	2.6 (2.2–3.5)

Mean arterial pressure (MAP), cardiac index and norepinephrine dose, were not significantly different between patients with septic shock with acute kidney injury (AKI) or without AKI (*p* = 0.07, *p* = 0.24, *p* = 0.13 for factor group with analysis of variance (ANOVA) on ranks). Lactate level was significantly higher in patients with septic shock with AKI than in patients with septic shock without AKI (**p = 0.008). The resistivity index was significantly higher in patients with septic shock with AKI than in patients with septic shock without AKI (*0.03 for group and 0.04 for interaction (time × group) with ANOVA on ranks). Mean transit time (mTT) was significantly lower in patients with septic shock without AKI than in patients with septic shock with AKI (^§§^*p* = 0.005 for group with ANOVA on ranks).

*AKI+* Kidney Disease Improving Global Outcomes (KDIGO) stage 2 or 3, *AKI-* no AKI or KDIGO stage 1, *CEUS* contrast enhanced ultrasound, *MAP* mean arterial pressure, *PI* perfusion index

### CEUS measurements

#### Comparison of septic shock and control groups

**Fig. 1:**
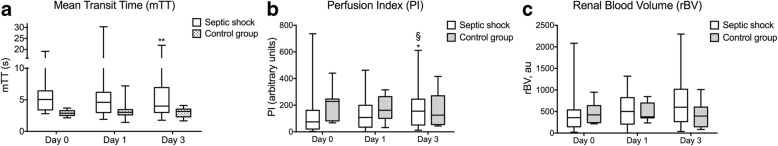
Evolution of mean transit time (mTT) (**a**), perfusion index (PI) (**b**) and rBV (**c**) during the first 3 days in ICU in patients with septic shock and in control patients without septic shock; a.u., arbitrary units. **p = 0.005 for group effect (control and septic shock groups) with analysis of variance (ANOVA) on ranks. *p = 0.03 for interaction (group × time) with ANOVA on ranks. ^§^*p* = 0.04 for ANOVA on ranks in the septic shock group over time. rBV ANOVA (*p* = 0.21). Box plots show median, interquartile (1;3) and maximum values

mTT (mean transit time) and PI (perfusion index) values were either higher, comparable or lower in patients with septic shock than in the control group (ICU patients without septic shock). Indeed, mTT values ranged from 2.8 to 19 s in the septic shock group (median 5.1 s (3.4–6.1) at day 0) and ranged from 2.2 to 3.7 s in ICU patients without septic shock (median 2.9 (2.6–3.1) at day 0) (Fig. 1a, day 0). Patients with septic shock had a wider range of PI values (3–736 a.u., median 74 (22–120) at day 0) than the control group (67–440 a.u., median 228 (103–235) at day 0) (Fig. 1b, day 0). rBV values ranged from 19 to 2084 a.u. in the septic shock group with median 356 (217–526) at day 0 (Fig. 1c, day 0). rBV values ranged from 215 to 947 a.u. in the control group with a median value of 423 (256–600) at day 0 (Fig. 1c, day 0).

mTT was significantly increased in the septic shock group (group effect *p* = 0.005) and PI was significantly decreased in the septic shock group (interaction group × time *p* = 0.03) compared to the control group (Fig. 1a and b). rBV was not significantly different between these two groups (Fig. 1c, *p* = 0.21).

PI improved significantly over time in the septic shock group (*p* = 0.04) (Fig. 1). In a post hoc analysis, PI at day 3 was higher than PI at day 0 (74 (22–120) versus 160 (88–245) *p* = 0.02). This improvement was not observed for mTT (*p* = 0.11).

#### Comparison of patients with septic shock with and without AKI

**Fig. 2:**
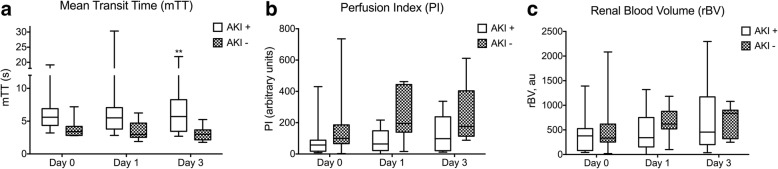
Evolution of mean transit time (mTT) (**a**), perfusion index (PI) (**b**) and rBV (**c**) during the first 3 days in ICU in patients with septic shock with acute kidney injury (AKI) Kidney Disease Improving Global Outcome (KDIGO) stage 2 or 3 (AKI+) or in patients with septic shock without AKI or with AKI KDIGO stage 1 (AKI-); a.u., arbitrary units. **p = 0.005 for group effect (with AKI or without AKI) with analysis of variance (ANOVA) on ranks. rBV ANOVA (*p* = 0.49). Box plots show median, interquartile (1;3) and maximum values

MAP, cardiac index, norepinephrine dose and lactate in patients with septic shock with AKI (KDIGO stage 2 or 3) and no AKI (no AKI or KDIGO stage 1) are given in Table 3. Patients with septic shock with AKI were older than patients with septic shock with no AKI (79 (70–86) vs 48 (37–56) respectively, *p* = 0.002) and had slightly worse chronic kidney disease (CKD) than patients with septic shock and no AKI (median glomerular filtration rate (GFR) 70 (55–84) and 94 (88–109) mL/min respectively, *p* = 0.01). CEUS measurements were compared between patients with septic shock with severe AKI (KDIGO stages 2 or 3) and no AKI (no AKI or KDIGO stage 1) (Fig. 2). Patients were allocated severe AKI or no AKI status according to their worst AKI stage in the first 72 h. mTT was significantly higher in patients with severe AKI compared with patients with no AKI over time (*p* = 0.005). No difference was observed in PI and rBV values between patients with severe AKI and patients with no AKI over time (*p* = 0.29 and *p* = 0.49, respectively). MAP, cardiac index and norepinephrine infusion rate were not significantly different between patients with septic shock with severe AKI and patients with septic shock with no AKI, whereas lactate concentration was significantly higher in patients with septic shock with severe AKI than in septic patients with no AKI (Table 3). Doppler RI was higher in the septic shock group with severe AKI than in the septic shock group with no AKI (Table 3).

**Fig. 3:**
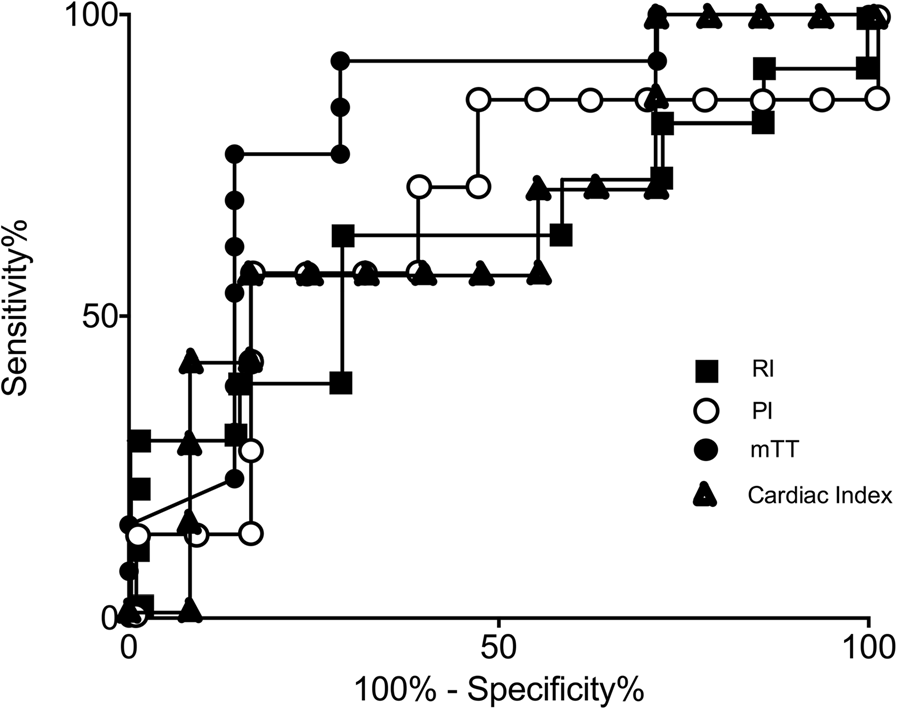
Receiver operating characteristic curves for prediction of severe acute kidney injury ( Kidney Disease Improving Global Outcome (KDIGO) stage 2 or 3) during the first 72 h with microhemodynamic data (mean transit time measured with contrast enhanced ultrasound (mTT), perfusion index measured with contrast enhanced ultrasound (PI), resistivity index measured with Doppler ultrasonography (RI)) and macrohemodynamic data (cardiac index)

The mTT, PI and rBV values (*p* = 0.06, *p* = 0.08 and *p* = 0.22, respectively) were not significantly different between ICU survivors and ICU non survivors in the septic shock group (data not shown). mTT at day 0 had the highest predictive value of severe AKI (AUC = 0.82 (0.60–1.04)) compared to RI, cardiac index and PI (AUC = 0.65 (0.41–0.90), AUC = 0.67(0.41–0.93) and AUC = 0.67 (0.40–0.94), respectively) (Fig. 3).

We used a linear multiple regression model to explore the association between microhemodynamic data (RI, glomerular filtration rate), macrohemodynamic data (MAP, cardiac index), metabolic data (lactate) and cortical renal perfusion (mTT, PI). Doppler RI and GFR were independently associated with mTT (Table 4). Doppler RI and cardiac index were independently associated with PI (Table 4).

**Table 4:** Coefficient values of multiple linear regression model with CEUS parameters

Dependent variables	MAP	*p*	CI	*p*	Lactate	*p*	Resistivity index	*p*	Creatinine clearance	*p*
PI	0.33	0.72	33.33	0.045	−9.23	0.28	− 574	< 0.001	0.171	0.43
mTT	0.026	0.36	0.485	0.34	0.065	0.80	13.14	0.009	−0.016	0.02

In the regression model, mean transit time (mTT) or perfusion index (PI) are the dependent variables and macrohemodynamic (mean arterial pressure (MAP), cardiac index (CI and lactate) and microhemodynamic parameters (resistivity index and calculated creatinine clearance) are the explanatory variables

*CEUS* contrast enhanced ultrasound

## Discussion

We performed ultrasound assessment of cortical renal perfusion during the first 72 h of septic shock resuscitation, using contrast technology for the first time for this indication. The first finding of this study was the highly variable cortical renal perfusion in patients with septic shock, which was high, intermediate or low despite satisfactory macrocirculatory hemodynamics. Furthermore, we showed that perfusion of the renal cortex is significantly reduced in patients with septic shock developing severe AKI (KDIGO stages 2 or 3) compared to those who do not develop AKI and compared to a control group without septic shock. Finally, a gradual improvement in renal cortical perfusion was observed over the first 72 h in patients with septic shock.

In this study, we quantified the cortical renal perfusion with CEUS for the first time and reported a decrease in renal cortical perfusion in patients with septic shock compared to ICU patients without septic shock. This decrease in cortical renal perfusion was the greatest in the first 24 h and progressively improved over the next 3 days. Renal hypoperfusion can lead to a decrease in renal function and was even reported to induce early renal injuries despite a complete correction of hypoperfusion after fluid resuscitation [21]. Studies conducted in patients with septic shock, using magnetic resonance imaging (MRI) and in patients with vasoplegic shock after cardiac surgery with renal venous thermodilution have also demonstrated a decrease in average renal blood flow [7, 22]. On the contrary, recent experimental studies conducted in animal models of septic shock have demonstrated preserved, or even increased renal blood flow with a concomitant decrease in GFR [8, 23, 24]. Maldistribution of blood flow inside the renal cortex and the renal medulla, which subsequently leads to a drop in renal medulla partial pressure of oxygen (PO_2_), could explain this renal function impairment despite preserved blood flow [25]. In our study, we report a result that is consistent with the divergent results of these studies, as cortical renal perfusion ranged from almost no perfusion to hyperemic perfusion (even above the cortical renal perfusion observed in the control group) despite the fact that MAP was in the recommended target level in septic shock (> 65 mmHg) and cardiac output was comparable to that of ICU patients without septic shock. As a consequence, based on the macrohemodynamic data, the level of cortical renal perfusion cannot be predicted. Lactate level was higher in patients with septic shock with AKI than in those with no AKI or mild AKI, suggesting that renal microcirculatory impairment is likely to occur in patients with the most severe metabolic consequences of septic shock. Because renal perfusion patterns are heterogeneous in patients with septic shock, renal CEUS could thus be an interesting tool for early detection of those patients with severe renal hypoperfusion. The introduction of this potential new noninvasive research tool would help to monitor the effect of interventions designed to prevent or treat early renal dysfunction and AKI. This obviously needs to be investigated in clinical practice.

The evolution of renal CEUS parameters was not statistically significantly different between survivors and non survivors, though a tendency was observed. This could be due to the small number of patients in our study.

A decrease in cortical renal perfusion measured with CEUS (increase in mTT) was associated with severe AKI occurrence in patients with septic shock , and an increase in renal resistivity index (RI), as reported in previous studies [12, 26]. Moreover, the strong and independent correlation between mTT and RI and between PI and RI suggests that RI changes are associated with cortical renal perfusion variations. This is consistent with previous Doppler ultrasound studies (measuring RI) that have suggested renal microcirculatory dysfunction in critically ill patients [26–28]. However, in this study, mTT was a better predictor of severe AKI (KDIGO stage 2 or 3) at day 0 than RI. The evaluation of cortical renal perfusion with CEUS (mTT) may be more accurate than Doppler ultrasound (RI) without contrast in detecting renal perfusion impairment.

Studies measuring renal perfusion with CEUS [18] or MRI [7] have not explored the association between renal perfusion and renal function. For the first time, we report an association between cortical renal perfusion and the occurrence of severe AKI in patients with septic shock. The greater the alteration in mTT (increase in mTT) the higher the risk of severe AKI. The decrease in renal cortical perfusion could have favored severe AKI in our cohort. However, on the contrary, evolving or established AKI may also have led to intrarenal microcirculatory disorders that further altered mTT. Interestingly, in a recent review reporting the renal histologic changes in experimental studies in septic AKI over 8 years (1059 animals), acute tubular necrosis was reported in 17% of animals and was only observed in the presence of decreased renal blood flow, and preserved renal blood flow was not associated with acute tubular necrosis [29]. Though renal function was altered either in the case of preserved or decreased renal blood flow in experimental septic shock, it seems that these two conditions have different effects on renal integrity.

Several parameters are available when studying renal perfusion with CEUS. mTT deals with the time needed to enhance renal parenchyma after contrast product destruction like “a renal refill time”. Alteration in dynamic perfusion tests (arm occlusion test with near-infrared spectroscopy) were reported to be associated with microvascular dysfunction in septic shock [30]. We reported an independent association between mTT and GFR and renal RI, whereas MAP and CI were not statistically associated with mTT. Thus, mTT seems to be mostly linked to intrarenal hemodynamics. The perfusion index (rBV/mTT) includes rBV, a measure of maximum CEUS signal intensity that is more variable than time to replenishment. Indeed, rBV depends on (1) the amount of circulating microbubbles (the concentration of which may vary between vials according to a ratio up to 1.5 (manufacturer data)), (2) the distance between the ultrasound probe and the kidney, which varies according to patients’ fluid balance and abdominal thickness and (3) renal cortical perfusion. These multiple factors are likely to explain why rBV failed to give relevant information on kidney perfusion. PI was independently associated with RI and CI but with MAP and GFR. Thus, PI may provide less information about intrarenal hemodynamics than mTT. This could explain why mTT and PI may differ though they are both considered renal perfusion parameters. In the end, mTT could be a more accurate parameter for evaluating intrarenal hemodynamics.

There are several limitations in our study. There was a heterogeneity in the renal CEUS parameters that were measured in the patients of the control group who were devoid of AKI. Renal CEUS quantification was reported to be reproducible in healthy volunteers [15, 31]. Thus, the heterogeneity between individuals should be due to the acquisition conditions in the ICU: the depth of renal cortex according to the patient’s body mass index, the respiratory movements that may modify the acquisition ultrasound plane and the thickness and echogenicity of tissue that are modified over time because of fluid balance increase. This heterogeneity was reported in previous studies of ICU patients; however, changes in cortical renal perfusion were easily detected despite this heterogeneity [18, 19]. Another explanation for measurement heterogeneity may come from patient enrollment at different phases of AKI, which probably correspond to different phases of microcirculatory alterations in the course of septic shock. A second limit is that we only measured cortical perfusion while medullary perfusion would be relevant and complementary [25]. Further studies are needed to investigate medullary perfusion during septic shock. This could be achieved in a future protocol with the injection of a higher dose of contrast because the renal medulla is less enhanced than the cortex at baseline. A measure of renal blood flow could have been relevant to distinguish between a decrease in global renal perfusion and an alteration in blood distribution inside the kidney. However, renal venous thermodilution would have been difficult to maintain for 3 days, with subsequent risk of renal venous thrombosis. This is a single-center study including surgical patients with septic shock, therefore further studies are needed to extend the conclusions to other ICUs. This study began before the new definitions of septic shock were published [32]. That is the reason why we used the former definition; 14 patients out of 20 would have satisfied the criteria for septic shock with the new definition. Patients with septic shock with AKI were older and had worse baseline renal function than those without AKI. Thus, it cannot be excluded that CEUS alterations were present because of older age or decreased baseline renal function in patients with AKI. Further investigations are necessary to extract the data on the changes observed by CEUS that are due to age, basal renal function and sepsis. We acknowledge that the sample size is small and subsequently implies the need for validation in a larger population. A larger cohort of patients would have allowed us to separate patients into four categories (no AKI, stage 1, stage 2, stage 3 AKI) to investigate if increasing severity of AKI is associated with increasing disorders in renal perfusion.

## Conclusions

In conclusion, though hemodynamic macrovascular parameters were restored, the cortical renal perfusion can be decreased, normal or even increased during septic shock. We observed an average decrease in cortical renal perfusion in patients with septic shock compared to patients without septic shock. The decrease in cortical renal perfusion was associated with occurrence of severe AKI. The use of renal CEUS to guide renal perfusion resuscitation needs further investigation.

## Abbreviations

AKI: Acute kidney injury
ANOVA: Analysis of variance
AP-HP: Assistance Publique Hôpitaux de Paris
a.u.: Arbitrary units
AUC: Area under the curve
CEERB: Comité d’Evaluation de l’Ethique des projets de Recherche Biomédicale
CEUS: Contrast enhanced ultrasound
CKD: Chronic kidney disease
CL: Control
DICOM: Digital imaging and communication in medicine
GFR: Glomerular filtration rate
GHU: Groupe Hospitalier Universitaire
HR: Heart rate
ICU: Intensive care unit
IRB: International review board
KDIGO: Kidney Disease Improving Global Outcome
MAP: Mean arterial pressure
MRI: Magnetic resonance imaging
mTT: Mean transit time
PI: Perfusion index
RBF: Renal blood flow
rBV: Renal blood volume
RI: Resistivity index
ROC: Receiver operating characteristic
ROI: Region of interest
SAPS II: Simplified Acute Physiology Score
SOFA: Sequential Organ Failure assessment
SV: Stroke volume
TTE: Transthoracic echocardiography
VTI: Velocity-time
